# Normal-sized basal ganglia perivascular space related to motor phenotype in Parkinson freezers

**DOI:** 10.18632/aging.203343

**Published:** 2021-07-27

**Authors:** Wen Lv, Yumei Yue, Ting Shen, Xingyue Hu, Lili Chen, Fei Xie, Wenying Zhang, Baorong Zhang, Yaxing Gui, Hsin-Yi Lai, Fang Ba

**Affiliations:** 1 Department of Neurology, Sir Run Run Shaw Hospital, Zhejiang University School of Medicine, Hangzhou, China; 2 Interdisciplinary Institute of Neuroscience and Technology, Zhejiang University School of Medicine, Hangzhou, China; 3 Department of Neurology of the Second Affiliated Hospital, Key Laboratory of Medical Neurobiology of Zhejiang Province, Zhejiang University School of Medicine, Hangzhou, China; 4 College of Biomedical Engineering and Instrument Science, Key Laboratory for Biomedical Engineering of Ministry of Education, Zhejiang University, Hangzhou, China; 5 Division of Neurology, Department of Medicine, University of Alberta, Edmonton, AB, Canada

**Keywords:** Parkinson's disease, perivascular spaces, motor phenotype, basal ganglia, 7T MRI

## Abstract

Changes in basal ganglia (BG) perivascular spaces (PVSs) are related to motor and cognitive behaviors in Parkinson’s disease (PD). However, the correlation between the initial motor phenotype and PVSs distribution/burden in PD freezing of gait (FOG) remains unclear. In addition, the normal-sized PVSs (nPVSs) have not been well-studied. With high-resolution 7T-MRI, we studied nPVSs burden in BG, thalamus, midbrain and centrum semiovale. The numbers and volume of nPVSs were assessed in 10 healthy controls, 10 PD patients without FOG, 20 with FOG [10 tremor dominant (TD), 10 non-TD subtype]. Correlation analyses were further performed in relation to clinical parameters. In this proof of concept study, we found that the nPVS burden of bilateral and right BG were significantly higher in freezers. A negative correlation existed between the tremor score and BG-nPVSs count. A positive correlation existed between the levodopa equivalent daily dose and BG-nPVSs count. The nPVS burden correlated with the progression to FOG in PD, but the distribution and burden of nPVS differ in TD vs. non-TD subtypes. High resolution 7T-MRI is a sensitive and reliable tool to evaluate BG-nPVS, and may be a useful imaging marker for predicting gait impairment that may evolve into FOG in PD.

## INTRODUCTION

Freezing of gait (FOG) is a common symptom in the advanced stages of Parkinson’s disease (PD). FOG increases the risk of falls and fall-related injuries with devastating impact on the quality of life of individuals with PD, often triggering a downward spiral of frailty and leading to depression, social isolation, activity avoidance, and fear of falling [1–3]. While classically occurring in advanced PD, FOG and falls can be seen in earlier stages, particularly in individuals who suffer from the postural instability gait difficulty (PIGD) subtype, when compared to the tremor-dominant (TD) subtype [4–6].

The mechanism of FOG in PD has been intensively studied. The “interference model” describes function interruption between cortical structures and brainstem regions involved in gait control possibly contributing to FOG [7, 8]. Similarly, the “decoupling model of FOG” suggests that a breakdown in coupling between posture preparation by the supplemental motor area and step initiation by the motor cortex may be responsible for the “start hesitation” in FOG [9]. It has been suggested that FOG may be due to a failure to generate adequate amplitudes of the intended movement [10]. The anatomical basis might be the failure of structural and functional integrity in the locomotion control system. For example, the widespread white matter damage involving sensorimotor-related and extramotor pathways was reported in PD-FOG patients. Individuals with diffused small vessel disease can frequently manifest Parkinsonian symptoms, while neuroimaging demonstrates diffused white matter hyperintensities (WMH). In addition, more severe WMH was found in the PIGD subtype of PD [11–14]. Left temporal WMH is related to falls in idiopathic PD [15]. Taken together, the white matter integrity and the subcortical network [involving regions such as the basal ganglia (BG), the thalamus and the mesencephalic locomotion center] are essential to maintain gait and balance. When damaged, FOG and balance impairment can occur.

Perivascular spaces (PVSs) are pial-lined interstitial fluid-filled spaces surrounding the penetrating blood vessels, which are commonly observed in the BG, white matter centrum semiovale (CSO), midbrain and subcortical white matter regions [16, 17]. The mechanism of PVS dilation remains unclear. Previous studies have demonstrated that enlarged PVSs (ePVSs) are associated with normal aging [18, 19]; cerebral small vessel disease [20–23]; neurodegenerative diseases, such as Alzheimer's disease [18, 24, 25] and PD [16, 26, 27]; stroke [20, 28–30]; neuroinflammation; and demyelination [31, 32]. Within BG, ePVSs are closely associated with older age, cerebral atrophy, lacunar stroke and cognitive impairment [33–35]. In PD, ePVSs and the severity of the PVS in BG are related to the severity of motor symptoms [36, 37], cognitive dysfunction [27], and future cognitive decline in individuals with normal cognition [38].

Since ePVSs are correlated with PD motor and cognitive impairment, one can postulate that the distribution and volume of the normal-sized PVSs (nPVSs) may have certain clinical significance in PD. Previous studies have mainly focused on ePVSs due to limits in imaging resolution. NPVSs are typically invisible due to small size in the range of 0.13-0.96 mm [39]. Seven Tesla (7T) MRI, with increased spatial resolution and signal-to-noise ratio, increases the detection of nPVSs [40, 41]. The 7T sequences have been optimized to provide detailed assessment of distributions of nPVSs in the white matter and subcortical nuclei [42].

In this proof of concept study, with 7T MRI, we investigated the clinical and neuroimaging significance of nPVS in important locomotion centers, including the BG, thalamus, midbrain, and CSO in PD freezers with different motor phenotypes. We hypothesized that the count and volume of nPVSs in BG may be different compared to those of age-matched healthy controls (HCs). The nPVSs burden of BG could potentially serve as a biomarker for PD gait impairment, and may further be a factor in distinguishing the motor subtypes in PD patients.

## RESULTS

### Demographic and clinical characteristics

The demographic and clinical characteristics of the HCs, PD patients without FOG [FOG(-)], PD patients with FOG tremor dominant subtype [FOG(TD)], and those with FOG, but non-TD type [FOG(TD-)] are shown in Table 1. There were no significant differences found in age, sex ratio, vascular risk factors, WMH burden and education level among the four groups. A majority of participants in the two FOG groups had moderate to severe degree of FOG (Table 1). Among the three PD groups, tremor score was significantly higher in the FOG(TD) group. The axial motor score, akinetic score, Levodopa equivalent daily dose (LEDD), Hamilton Depression Scale (HAMD) and Hamilton Anxiety Scale (HAMA) scores were higher in the freezers.

**Table 1:** Demographic and clinical characteristics of the participants.

	**HCs (n=10)**	**PD FOG(-) (n=10)**	**FOG(TD) (n=10)**	**FOG(TD-) (n=10)**	**P value^a^**	**P value^b^**
**Sex (M/F)**	5/5	5/5	2/8	6/4		0.33
**Age (years)**	61.36±4.40	66.27±4.69	65.23±4.92	66.23±5.05		0.09
**Hypertension, n**	7	6	3	5	0.40	
**Hyperlipidemia, n**	1	0	2	0	0.60	
**Diabetes Mellitus, n**	1	0	0	2	0.60	
**Stroke, n**	0	0	0	0		
**Cardiac disease, n**	0	0	0	0		
**Cigarette, n**	3	2	0	1	0.46	
**WMH score**	3.50±2.80	4.70±1.95	4.80±2.49	5.20±4.29	0.62	
**Disease duration (years)**	NA	8.20±6.09	11.50±6.34	9.40±2.67	0.38	
**FOG duration (years)**	NA	NA	3.18±3.53	2.70±2.75	0.34	
**UPDRS total**	NA	51.70±18.51	61.30±15.76	63.40±14.37	0.25	
**UPDRS-III (OFF)**	NA	36.67±10.79	38.00±3.16	42.25±5.42	0.25	
**UPDRS-III(ON)**	NA	29.00±3.61	27.50±5.74	26.50±6.61	0.83	
**Improvement (%)**	NA	18.25±14.74	27.90±11.96	35.44±21.22	0.40	
**Tremor score**	NA	6.00±4.59	7.60±4.70	1.70±1.34	<0.01*	
**Axial motor score (OFF)**	NA	4.50±2.07	9.25±5.06	9.67±3.00	<0.01*	
**Axial motor score (ON)**	NA		5.50±1.93	6.00±2.83	0.66	
**Improvement (%)**	NA		40.54±36.91	38.43±21.03	0.83	
**Rigidity score**	NA	7.30±3.37	7.80±1.62	8.70±4.27	0.63	
**Akinetic score**	NA	13.10±5.22	13.10±5.00	17.70±2.98	<0.05*	
**Akinetic-Rigid score**	NA	20.40±7.59	20.90±5.99	26.40±6.19	0.10	
**NFOGQ score**	NA	0	23.50±3.17	20.70±7.15	0.01*	
**LEDD (mg/day)**	NA	506.95±299.24	647.45±256.15	841.55±311.66	0.04*	
**MMSE score**	26.70±2.87	23.60±4.33	20.90±6.23	21.00±6.25		<0.05*
**HAMD score**	2.80±2.35	6.70±5.14	12.60±923	9.00±6.38		<0.01*
**HAMA score**	2.40±2.37	9.90±7.25	10.70±4.19	8.50±5.62		0.01*

Data were presented as mean ± SD. FOG, Freezing of Gait; FOG(T-), FOG without tremor; FOG(T+), FOG with tremor; PD-FOG (-), PD without FOG; UPDRS, Unified Parkinson's Disease Rating Scale; NFOGQ, New Freezing of Gait Questionnaire; LEDD, Levodopa Equivalent Daily Dose; MMSE, Mini-Mental State Examination; HAMD, Hamilton Depression Scale; HAMA, Hamilton Anxiety Scale, NA, Not Applicable.

Tremor score, the sum of UPDRS item 16 (arms tremor identified by history), 20 (face and four limbs tremor at rest), 21 (arms action or postural tremor); Axial motor score, the sum of UPDRS item 13 (falling), 14 (freezing), 15 (walking), 29 (gait), 30 (postural instability); Rigidity score, UPDRS item 22 (rigidity of neck and four limbs); Akinetic score, the sum of UPDRS item 23 (finger tap), 24 (hand movement), 25 (hand rotation), 26 (feet flexibility), 31 (body bradykinesia), akinetic-rigid score (sum of items 22–26 and 31).*, p<0.05. A 30% improvement in UPDRS-III was considered good levodopa response with levodopa challenge.

a. Comparison among PD groups.

b. Comparison among PD groups and age-matched healthy controls.

### Analysis of the nPVSs in basal ganglia

**Figure 1:**
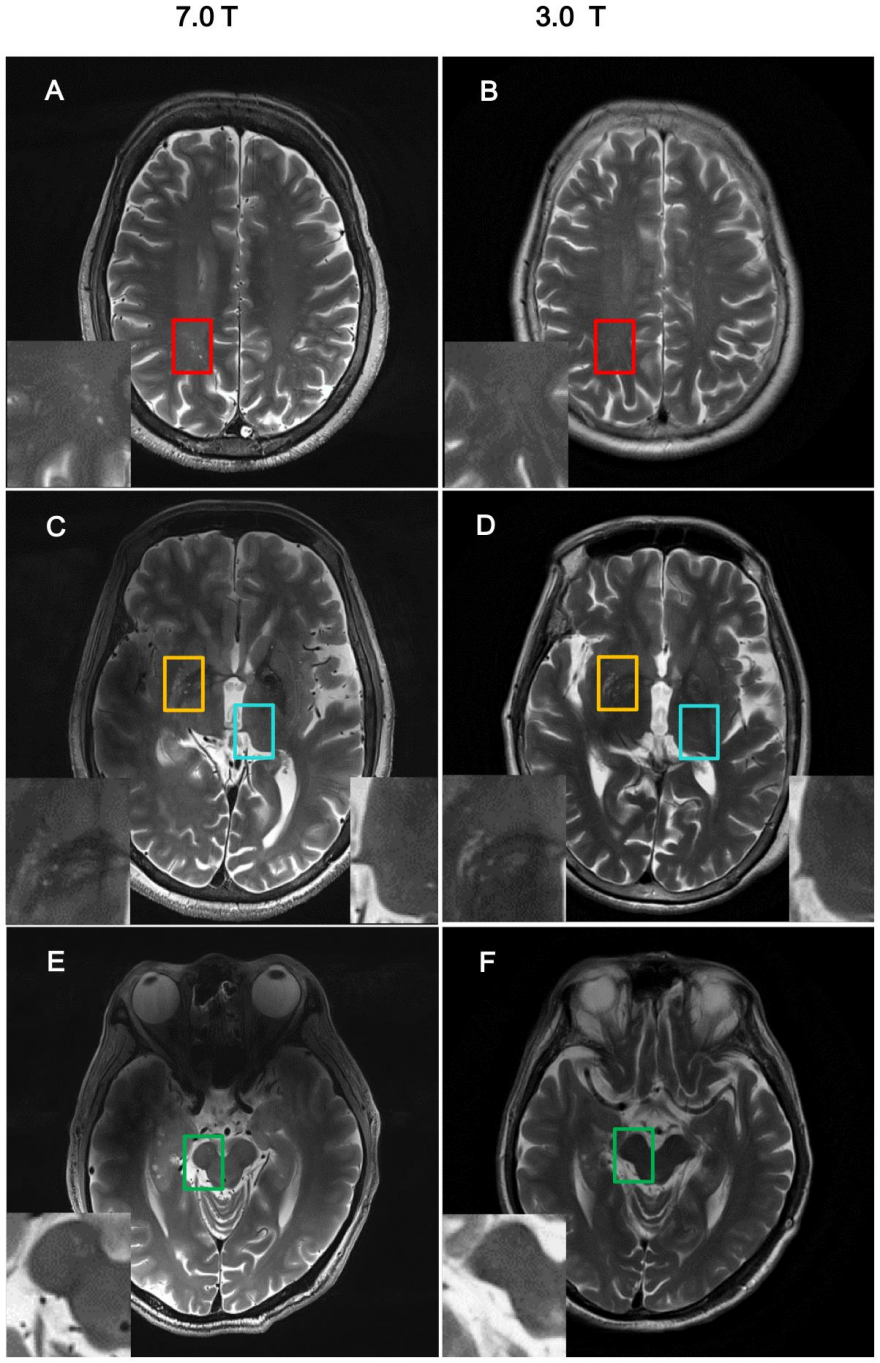
**Comparison of imaging resolution between 7T and 3T MRI for nPVS.** Example of comparisons of resolution of nPVSs on T2 weighted images acquired by 7.0T MRI vs 3.0T MRI on the same study participant. (**A**, **B**) Indicate centrum semiovale with red square; (**C**, **D**) indicate yellow squares for basal ganglia and blue squares for thalamus; (**E**, **F**) is midbrain with green squares. nPVS, normal-sized perivascular space.

With 7T MRI, the resolution of the images was high enough to allow analysis of nPVS burden (Figure 1). NPVS number and volume calculation of PD subgroups and HCs groups were performed (Table 2). Test-retest reliability using the two-way mixed model for absolute agreement over a one-month interval reached 0.79 and 0.80 for nPVSs number and volume of BG region, 0.72 and 0.74 of thalamic region, 0.89 and 0.93 for the CSO region, and 0.77 and 0.83 of the midbrain, respectively.

**Table 2:** NPVSs count and volume in basal ganglia.

	**HCs**	**FOG(-)**	**FOG(TD)**	**FOG(TD-)**	**p-value**
**nPVSs count**					
Left	6.30 ± 0.99	8.50 ± 1.20	9.50 ± 1.46	11.90 ± 2.11	0.08
Right	6.20 ± 0.57	9.60 ± 1.77	13.20 ± 1.78	18.70 ± 2.11	<0.001*
Bilateral	12.50 ± 1.18	18.10 ± 2.27	22.70 ± 2.15	30.60 ±3.56	<0.001*
Slice with highest count	7.70 ±0.60	11.60 ± 1.42	13.80 ± 1.65	19.10 ± 2.01	<0.001*
**nPVSs volume (mm^3^)**					
Left	74.26 ±8.54	52.27±6.14	81.48±8.53	89.07±9.57	0.02*
Right	57.41±8.39	54.12±6.48	65.02±11.57	99.73±13.33	0.03*
Bilateral	131.67±15.29	106.40±10.09	146.49±17.46	188.79±20.73	0.01*
Slice with highest count	22.71 ± 3.71	24.40 ± 2.51	28.80 ± 4.12	40.51 ± 3.75	0.005*

Data were presented as mean ± SEM. nPVSs, normal-sized perivascular spaces; HCs, healthy controls; FOG, freezing of gait; FOG(-), PD patients without FOG; FOG(TD), PD patients with FOG whose motor phenotype was tremor dominant; FOG(TD-), PD patients with FOG whose motor phenotype was PIGD or indeterminate.

*, p<0.05, one way ANOVA.

And nPVS volume is calculated and presented as: left, unilateral slice with the highest number of nPVS at the left side and two slices below and above; right, unilateral slice with the highest number of nPVS at the right side and two slices below and above; bilateral, the sum of the above left and right; slice with highest count, nPVS analysis on the single slice with the highest number of nPVS bilaterally.

**Figure 2:**
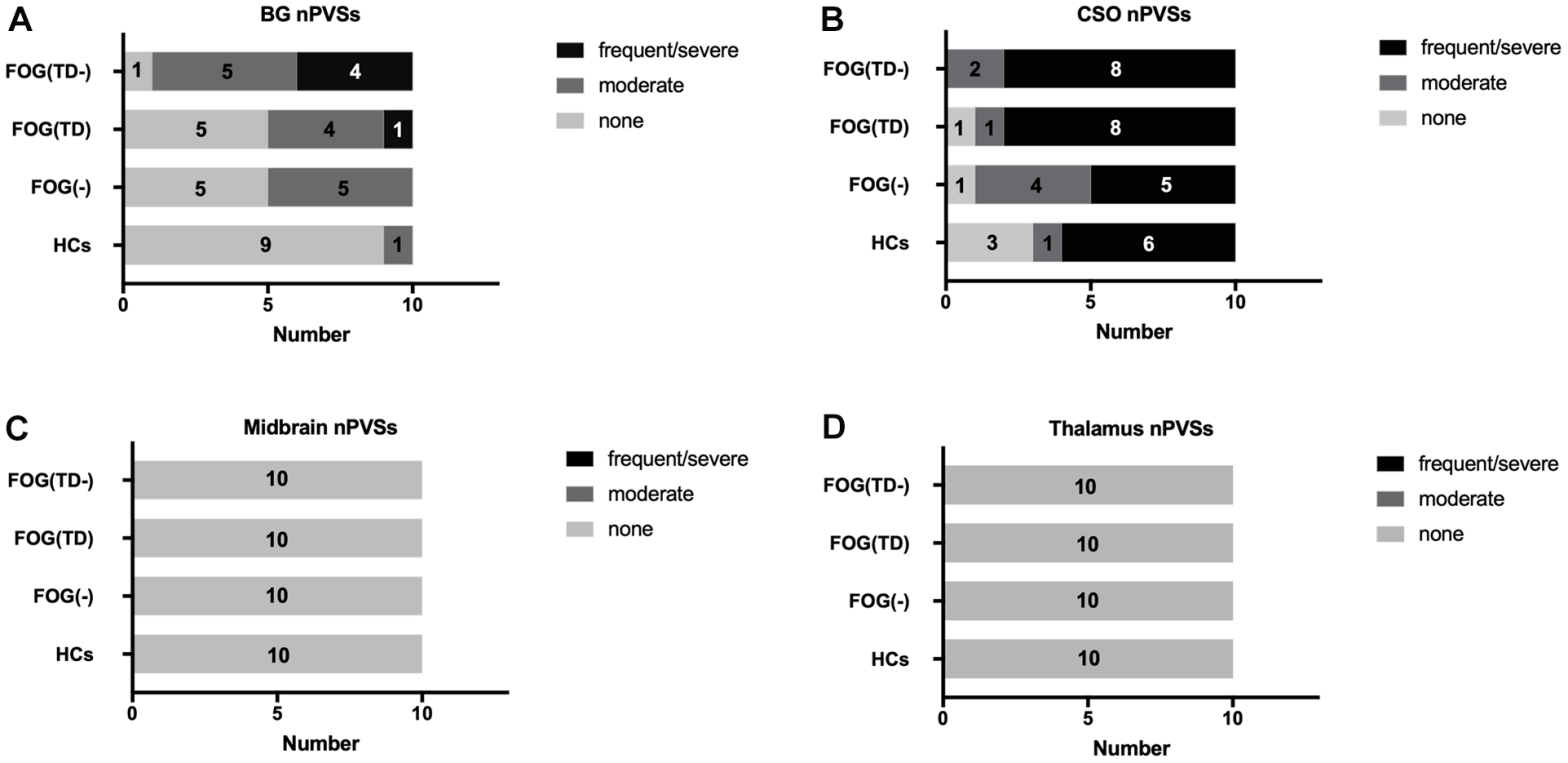
**Semi-quantitative assessment of nPVSs severity.** nPVS severity was assessed using a semi-quantitative scale (none/mild = 0/1, moderate = 2, frequent/severe = 3/4). The severity is shown in the basal ganglia (**A**), CSO (**B**), midbrain (**C**) and thalamus (**D**). nPVS, normal-sized perivascular space; BG, basal ganglia; CSO, centrum semiovale; FOG, freezing of gait; TD, tremor dominant; HCs, healthy controls; FOG(-), PD patients without FOG; FOG(TD), PD patients with FOG TD subtype; and FOG(TD-) PD patients with FOG, but non-TD subtypes.

The nPVS numbers of the right and bilateral BG were significantly higher in the FOG(TD-) group than the rest of the groups using one-way ANOVA (Table 2 and Figure 2). The volume of the nPVS of FOG(TD-) group was significantly higher than the other groups when compared unilaterally, bilaterally or choosing a single slice with the highest count (Table 2). No differences were detected in thalamus, CSO, or midbrain regions among the groups. No moderate or severe nPVS burden was seen in the thalamus or midbrain using the scale system previously described (Figure 2) [43].

### Correlation between BG-nPVS burden with clinical features and WMH burden

**Figure 3:**
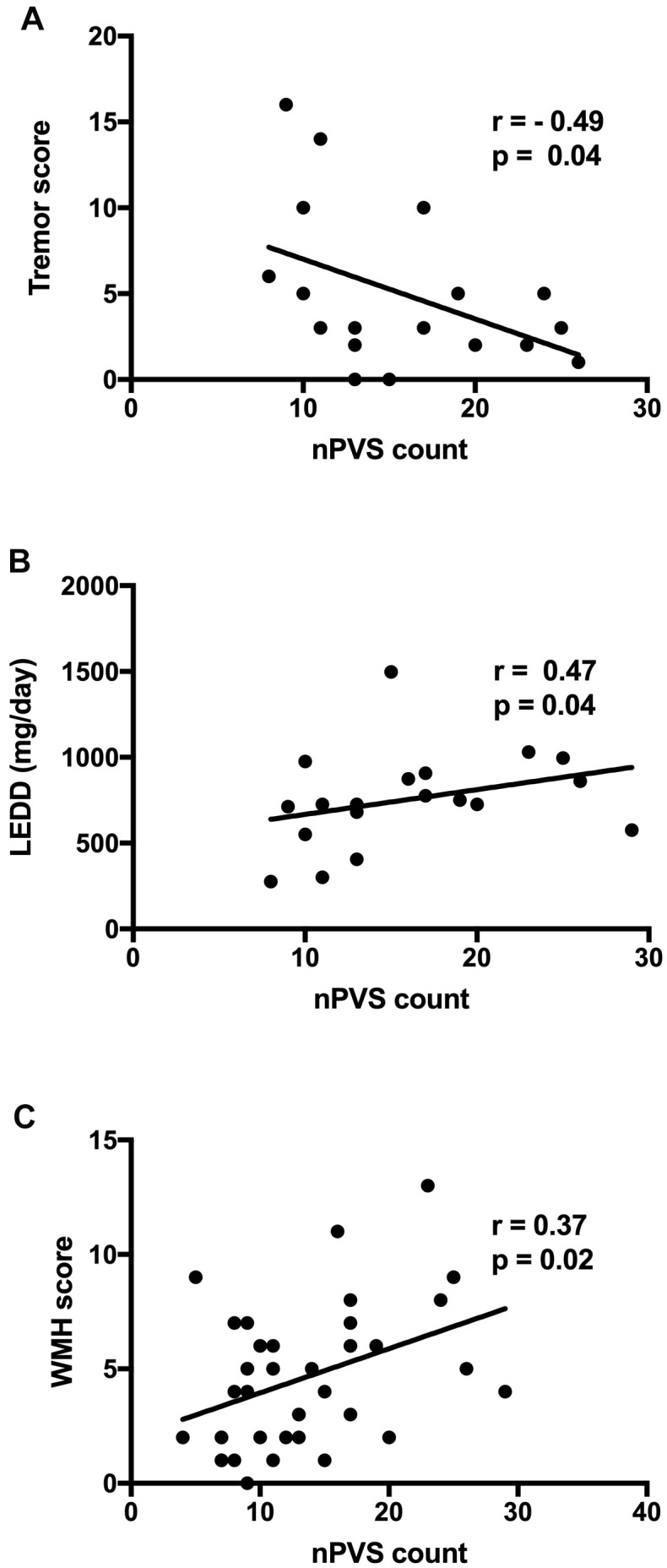
**Correlation between BG nPVS burden with clinical features and WMH score.** (**A**) Correlation between the tremor score and BG nPVSs count; (**B**) correlation between LEDD and nPVSs count of BG; and (**C**) correlation between WMH score and nPVSs count of BG. BG, basal ganglia; nPVs, normal-sized perivascular space; WMH, white matter hyperintensity; LEDD, levodopa equivalent daily dose.

In PD freezers, a significantly negative correlation existed between the tremor score and BG-nPVSs count (r = -0.49, p = 0.04, Figure 3A), and a positive correlation was found between the LEDD and nPVSs count of BG (r = 0.47, p = 0.04, Figure 3B). An overall positive correlation between WMH burden and BG-nPVS (r = 0.37, p = 0.02, Figure 3C) for all 40 participants was found. There were no correlations between nPVS burden and the UPDRS-III as well as other clinical parameters. There were no correlations between BG-nPVS volume and clinical parameters. There was no difference in the nPVS count and burden in the other areas assessed, nor was there any clinical correlation detected.

## DISCUSSION

In this proof of concept study, we investigated the utility of ultra-high field 7T MRI to assess nPVS burden and determine whether nPVS counts and volume could serve as imaging tools to distinguish motor phenotypes in PD freezers. First, we established that 7T MRI could be a reliable tool in assessing nPVS. The significance of normal sized nPVS in BG has not been well studied partially due to the challenges associated with nPVS quantitation using lower resolution MRI scanners. Conversely, using a 7T MRI scanner with the higher field strength makes it possible to quantitate nPVSs.

PVSs are microscopic but visible on MRI when enlarged with the widely used 1.5 and 3T scanner. PVSs are commonly seen in healthy adults, in BG and CSO in up to 60% of individuals [44]. There is clinical relevance to PVS. PVSs that relate to small vessel diseases are contributing factors to stroke and dementia [45, 46]. It has also been proposed that ePVS is relevant to the development of neurodegenerative disease [47]. In PD patients, periventricular WMH, brain atrophy, and BG-ePVSs have been noted to impact motor and cognitive functions [16, 26]. A previous study has shown that vascular factors might be involved in the pathophysiology of PIGD motor phenotype [48]. Postural and gait control involves integration of sensorimotor, BG, thalamus and cerebellum circuitries [49]. A recent study exploring the association between small-vessel diseases and motor symptoms of PD showed different clinical association. A close association between ePVS in BG and the tremor score, as well as between deep WMH and the axial motor score were seen [50]. However, this study did not explore the correlations with FOG.

The current study demonstrated a link between motor phenotypes and BG-nPVS burden. We first showed that nPVS burden in the BG was significantly higher in PD patients with FOG than those without FOG and the control group. The nPVS burden was significantly higher in right BG and bilateral BG among the PD freezers. Lateralization of the structural and functional connectivities in the human brain was reported in multiple studies of FOG, and it was noted that FOG was strongly related to structural deficits in the right hemisphere’s locomotor network [51–54]. Right hemisphere PD pathology has been associated with more impairments in multiple cognitive domains, including verbal recall, semantic verbal fluency, visuospatial analysis, and attention span [55]; it is also related to slower gait [56] and poorer axial mobility [57]. Functional connectivity was reduced within the executive-attention network in FOG patients within the right middle frontal gyrus [58]. In our study, it is hard to conclude whether the lateralization is significant due to the small sample size.

We observed a less severe nPVS burden with the initial motor phenotype being TD subtype than the non-TD subtypes in PD freezers. The negative correlation between the tremor score and the nPVS number of BG may partially explain why the TD subtype carries a better prognosis. Response to levodopa therapy differs in PD subtypes, and it is known that axial symptoms, i.e. gait and balance tend to be less responsive to dopaminergic agents [59, 60]. The higher LEDD dose in the freezers and the positive relationship between LEDD and BG-nPVS number are consistent with the previous observations that poorer levodopa response occurs when higher damage to the neurocircuitry is evident in the PIGD subtype.

We have shown a positive correlation between WMH burden and BG-nPVS. Given the known correlation between WMH and gait deficit in PD [11–14], and the evolving evidence of BG-ePVSs and motor symptoms [36, 37], and cognitive dysfunction [27, 38] in PD, our study suggested that increased nPVS in the BG region may act as a biomarker of gait decline if this finding holds in a larger study. Whether such changes relate to disruptions of the neural circuitry for gait control warrants further investigations with structural and functional connectivity studies. There was no association between CSO nPVS burden and PD motor symptoms, which is consistent with previous studies that the severity of axial motor impairments was not associated with the intensity of the periventricular WMH, suggesting certain functional distinctions between BG PVS and CSO PVS [61, 62]. Although not well studied, nPVS distribution and burden may also reflect the similar degenerative processes with ePVS. The advance in recent imaging technologies make it possible to assess such microstructural changes *in vivo*, especially with high-field MRI scanners*.* Such assessment in relation to clinical parameters can potentially serve as biomarkers to monitor disease progression and more precisely differentiate disease phenotypes.

The strengths of our study include application of a novel tool to assess a potential imaging marker for PD. Although the literature on PVS in PD are growing, and there are more evidence to show the link between higher BG PVS burden and future cognitive decline [38] and motor manifestations [36]; using high resolution 7T MRI to compare the distribution and volume of nPVS in BG, and identifying how these parameters correlate with motor phenotype in PD is novel. We established a method and identified the role of nPVS in a specific group of PD patients, with a focus on the most disabling motor symptom, FOG. With technology advancing rapidly, building on knowledge and expertise with better imaging tools will aid further development in the field. We speculate that the research work with 7T MRI scanners will bring new insights, and soon add new knowledge to clinical practice. This proof of concept study encourages further investigation in future large-scale studies when 7T MRI scanners are more readily available. There are some limitations. This is a single-centered proof of concept study with relatively small sample size. Further, this study has a focus on FOG since it is one of the most disabling symptoms in PD and the mechanism is not fully clear. Due to these factors, we cannot extrapolate the findings to all PD patients, or explore the sex differences. Future large prospective studies will provide more insight to further investigate the utility of 7T MRI in evaluating nPVS as an imaging biomarker for disease phenotyping and trajectory.

## CONCLUSIONS

We proposed a method using a high resolution 7T MRI to evaluate nPVS in BG to provide a potential imaging marker for predicting gait impairment in PD. The current study demonstrates that the nPVS burden correlates with the progression to FOG in PD patients, but the distribution and burden of nPVS may differ in people with or without tremor as initial motor presentation. High resolution 7T MRI is a sensitive and reliable tool to evaluate BG-nPVS, and may be a useful imaging marker for predicting gait impairment that may evolve into FOG in PD.

## MATERIALS AND METHODS

### Study participants

Twenty PD patients with FOG, 10 FOG(TD), 10 FOG(TD-), 10 PD(FOG-), and 10 age- and sex-matched HCs were recruited from the Department of Neurology of Sir Run Run Shaw Hospital (Table 1). The study was approved by the ethic committee of Sir Run Run Shaw Hospital of Zhejiang University School of Medicine (Ethics No. 20200908-30). All patients were diagnosed with PD by a movement disorders neurologist based on the UK Parkinson’s Disease Society Brain Bank criteria [63], and FOG was defined as a score of one or more on item 3 of the New FOG questionnaire (NFOG-Q) [64] or by history and examination by two experienced movement disorders neurologists. All participants were examined by experienced neurologists with a full neurological examination. Patients with gait issues secondary to visual impairments, sensory ataxia, and orthopedic issues were excluded. We also excluded patients with atypical Parkinsonism. All participants with moderate to significant small vessel disease were excluded, and HCs reported no history of neurological or psychiatric disorders. Clinical assessment included Unified Parkinson’s Disease Rating Scale (UPDRS) for PD motor symptoms and NFOG-Q for FOG severity, respectively. Cognitive function and mental health were evaluated using Mini Mental State Examination (MMSE), HAMD and HAMA. LEDD was calculated [65]. Other inclusion criteria of the study included disease duration ≥ 5 years, and Hoehn-Yahr stage < 4. Patients with significant cognitive deficits that prevent them from signing consent, and motor symptoms that were secondary to other etiologies were excluded. Based on the initial motor phenotypes, PD-FOG patients were divided into two groups, FOG(TD) and FOG(TD-) (PIGD and indeterminate) [4]. Patients’ motor function was assessed during the defined off-medication state after dopaminergic medications were on hold for more than 12 hours. Repeat motor examination was performed after patients reported ON with a supra-ON dose of LED (150% of the regular morning dose in Levodopa/carbidopa formula). Cognitive examination and other questionnaires were acquired during ON state.

### MR imaging acquisition and analysis

MR images were acquired with a 7T Magnatom research system (Siemens Healthcare, Erlangen, Germany) with a T2-weighted turbo spin echo (TSE) sequence (voxel size: 0.5 × 0.5 × 2.4 mm^3^, TR = 7000 ms, and TE = 66 ms), and two rapid gradient echoes (MP2RAGE) sequence (voxel size: 0.7 × 0.7 × 0.7 mm^3^, TR = 5000 ms, TI1/TI2 = 900/2750ms, TE = 2.3 ms, α1/α2 = 5°/3°). The nPVS burden, nPVS number and volume, were calculated with the axial T2-weighted TSE images via ITK-SNAP Software version 3.8 (http://www.itksnap.org/) by a neurologist blinded to the participant’s diagnosis and clinical features. For nPVS volume assessment, the border of each nPVS in the chosen slice was drawn manually. The nPVS volumes within the border were calculated automatically by the toolbox. The number of nPVSs were counted in the BG, thalamus, midbrain and CSO regions (Figure 1). For BG, thalamus, and CSO, nPVSs were assessed on the slice unilaterally with the highest number for left or right side, followed by the sum of both sides. We then assess a single slice with the highest total nPVS count. For midbrain, given it is a small structure, nPVSs were counted within all slices showing midbrain. A 4-point visual rating scale (0 = no PVSs, 1 = PVSs < 10, 2 = 11-20 PVSs, 3 = 21-40 PVSs, 4 = PVSs > 40) were used to grade the severity of PVS [66]. PVSs severity was then assessed using a semi-quantitative scale (none/mild = 0/1, moderate = 2, frequent/severe = 3/4) [43]. All patients were included for test-retest reliability testing. The WMH burden for all participants was assessed by using a semi-quantitative rating scale [67].

### Statistical analysis

Statistical analysis was performed with SPSS statistics (Version 22, IBM Corporation, Armonk, NY, USA). Categorial variables were analyzed with Fisher’s exact test. Continuous variables were analyzed with One-way ANOVA. Correlation analyses between nPVS burdens, nPVS number and volume, and clinical features, namely MMSE, HAMA, HAMD, UPDRS-III and LEDD, were conducted using spearman correlation analysis. In addition, we also analyzed the correlation between nPVS number of BG and WMH burden. *P* < 0.05 was considered to define statistical significance.

With SPSS, intra-class correlation coefficients (ICC) was calculated. The ICC analysis assessed the test-retest reliability via the 2-way mixed model for absolute agreement. It was defined that ICC between 0.60-0.74 as good, and above 0.75 being excellent.

## References

[1] Perez-Lloret S, Negre-Pages L, Damier P, Delval A, Derkinderen P, Destée A, Meissner WG, Schelosky L, Tison F, Rascol O. Prevalence, determinants, and effect on quality of life of freezing of gait in Parkinson disease. JAMA Neurol. 2014; 71:884–90. 10.1001/jamaneurol.2014.753 24839938

[2] Bloem BR, Hausdorff JM, Visser JE, Giladi N. Falls and freezing of gait in Parkinson’s disease: a review of two interconnected, episodic phenomena. Mov Disord. 2004; 19:871–84. 10.1002/mds.20115 15300651

[3] Factor SA. The clinical spectrum of freezing of gait in atypical parkinsonism. Mov Disord. 2008 (Suppl 2); 23:S431–38. 10.1002/mds.21849 18668624

[4] Jankovic J, McDermott M, Carter J, Gauthier S, Goetz C, Golbe L, Huber S, Koller W, Olanow C, Shoulson I, Stern M, Tanner C, Weiner W, and The Parkinson Study Group. Variable expression of Parkinson’s disease: a base-line analysis of the DATATOP cohort. The Parkinson Study Group. Neurology. 1990; 40:1529–34. 10.1212/wnl.40.10.1529 2215943

[5] Macht M, Kaussner Y, Möller JC, Stiasny-Kolster K, Eggert KM, Krüger HP, Ellgring H. Predictors of freezing in Parkinson’s disease: a survey of 6,620 patients. Mov Disord. 2007; 22:953–56. 10.1002/mds.21458 17377927

[6] Giladi N, McDermott MP, Fahn S, Przedborski S, Jankovic J, Stern M, Tanner C, and Parkinson Study Group. Freezing of gait in PD: prospective assessment in the DATATOP cohort. Neurology. 2001; 56:1712–21. 10.1212/wnl.56.12.1712 11425939

[7] Lewis SJ, Barker RA. A pathophysiological model of freezing of gait in Parkinson’s disease. Parkinsonism Relat Disord. 2009; 15:333–38. 10.1016/j.parkreldis.2008.08.006 18930430

[8] Nieuwboer A, Giladi N. Characterizing freezing of gait in Parkinson’s disease: models of an episodic phenomenon. Mov Disord. 2013; 28:1509–19. 10.1002/mds.25683 24132839

[9] Jacobs JV, Nutt JG, Carlson-Kuhta P, Stephens M, Horak FB. Knee trembling during freezing of gait represents multiple anticipatory postural adjustments. Exp Neurol. 2009; 215:334–41. 10.1016/j.expneurol.2008.10.019 19061889 PMC3141813

[10] Chee R, Murphy A, Danoudis M, Georgiou-Karistianis N, Iansek R. Gait freezing in Parkinson’s disease and the stride length sequence effect interaction. Brain. 2009; 132:2151–60. 10.1093/brain/awp053 19433440

[11] Lee SJ, Kim JS, Lee KS, An JY, Kim W, Kim YI, Kim BS, Jung SL. The severity of leukoaraiosis correlates with the clinical phenotype of Parkinson’s disease. Arch Gerontol Geriatr. 2009; 49:255–59. 10.1016/j.archger.2008.09.005 18977043

[12] Bohnen NI, Müller ML, Zarzhevsky N, Koeppe RA, Bogan CW, Kilbourn MR, Frey KA, Albin RL. Leucoaraiosis, nigrostriatal denervation and motor symptoms in Parkinson’s disease. Brain. 2011; 134:2358–65. 10.1093/brain/awr139 21653540 PMC3155702

[13] Piccini P, Pavese N, Canapicchi R, Paoli C, Del Dotto P, Puglioli M, Rossi G, Bonuccelli U. White matter hyperintensities in Parkinson’s disease. Clinical correlations. Arch Neurol. 1995; 52:191–94. 10.1001/archneur.1995.00540260097023 7848130

[14] Sohn YH, Kim JS. The influence of white matter hyperintensities on the clinical features of Parkinson’s disease. Yonsei Med J. 1998; 39:50–55. 10.3349/ymj.1998.39.1.50 9529985

[15] Ciliz M, Sartor J, Lindig T, Pilotto A, Schäffer E, Weiss M, Scheltens P, Becker S, Hobert MA, Berg D, Liepelt-Scarfone I, Maetzler W. Brain-Area Specific White Matter Hyperintensities: Associations to Falls in Parkinson’s Disease. J Parkinsons Dis. 2018; 8:455–62. 10.3233/JPD-181351 30040742

[16] Groeschel S, Chong WK, Surtees R, Hanefeld F. Virchow-Robin spaces on magnetic resonance images: normative data, their dilatation, and a review of the literature. Neuroradiology. 2006; 48:745–54. 10.1007/s00234-006-0112-1 16896908

[17] Kwee RM, Kwee TC. Virchow-Robin spaces at MR imaging. Radiographics. 2007; 27:1071–86. 10.1148/rg.274065722 17620468

[18] Guo H, Siu W, D’Arcy RC, Black SE, Grajauskas LA, Singh S, Zhang Y, Rockwood K, Song X. MRI assessment of whole-brain structural changes in aging. Clin Interv Aging. 2017; 12:1251–70. 10.2147/CIA.S139515 28848333 PMC5557118

[19] Grajauskas LA, Siu W, Medvedev G, Guo H, D’Arcy RC, Song X. MRI-based evaluation of structural degeneration in the ageing brain: Pathophysiology and assessment. Ageing Res Rev. 2019; 49:67–82. 10.1016/j.arr.2018.11.004 30472216

[20] Duperron MG, Tzourio C, Schilling S, Zhu YC, Soumaré A, Mazoyer B, Debette S. High dilated perivascular space burden: a new MRI marker for risk of intracerebral hemorrhage. Neurobiol Aging. 2019; 84:158–65. 10.1016/j.neurobiolaging.2019.08.031 31629114

[21] Zhai FF, Ye YC, Chen SY, Ding FM, Han F, Yang XL, Wang Q, Zhou LX, Ni J, Yao M, Li ML, Jin ZY, Cui LY, et al. Arterial Stiffness and Cerebral Small Vessel Disease. Front Neurol. 2018; 9:723. 10.3389/fneur.2018.00723 30210443 PMC6121106

[22] Ishikawa M, Yamada S, Yamamoto K. Dilated Perivascular Spaces in the Centrum Semiovale Begin to Develop in Middle Age. J Alzheimers Dis. 2018; 61:1619–26. 10.3233/JAD-170755 29376866

[23] Duperron MG, Tzourio C, Sargurupremraj M, Mazoyer B, Soumaré A, Schilling S, Amouyel P, Chauhan G, Zhu YC, Debette S. Burden of Dilated Perivascular Spaces, an Emerging Marker of Cerebral Small Vessel Disease, Is Highly Heritable. Stroke. 2018; 49:282–87. 10.1161/STROKEAHA.117.019309 29311265

[24] Hansen TP, Cain J, Thomas O, Jackson A. Dilated perivascular spaces in the Basal Ganglia are a biomarker of small-vessel disease in a very elderly population with dementia. AJNR Am J Neuroradiol. 2015; 36:893–98. 10.3174/ajnr.A4237 25698626 PMC7990588

[25] Roher AE, Kuo YM, Esh C, Knebel C, Weiss N, Kalback W, Luehrs DC, Childress JL, Beach TG, Weller RO, Kokjohn TA. Cortical and leptomeningeal cerebrovascular amyloid and white matter pathology in Alzheimer’s disease. Mol Med. 2003; 9:112–22. 12865947 PMC1430731

[26] 3C Study Group. Vascular factors and risk of dementia: design of the Three-City Study and baseline characteristics of the study population. Neuroepidemiology. 2003; 22:316–25. 10.1159/000072920 14598854

[27] Shibata K, Sugiura M, Nishimura Y, Sakura H. The effect of small vessel disease on motor and cognitive function in Parkinson’s disease. Clin Neurol Neurosurg. 2019; 182:58–62. 10.1016/j.clineuro.2019.04.029 31078957

[28] Thomas O, Cain J, Nasralla M, Jackson A. Aortic Pulsatility Propagates Intracranially and Correlates with Dilated Perivascular Spaces and Small Vessel Compliance. J Stroke Cerebrovasc Dis. 2019; 28:1252–60. 10.1016/j.jstrokecerebrovasdis.2019.01.020 30770255

[29] Puy L, Barbay M, Roussel M, Canaple S, Lamy C, Arnoux A, Leclercq C, Mas JL, Tasseel-Ponche S, Constans JM, Godefroy O, and GRECogVASC Study Group. Neuroimaging Determinants of Poststroke Cognitive Performance. Stroke. 2018; 49:2666–73. 10.1161/STROKEAHA.118.021981 30355190

[30] MacGregor Sharp M, Bulters D, Brandner S, Holton J, Verma A, Werring DJ, Carare RO. The fine anatomy of the perivascular compartment in the human brain: relevance to dilated perivascular spaces in cerebral amyloid angiopathy. Neuropathol Appl Neurobiol. 2019; 45:305–08. 10.1111/nan.12480 29486067

[31] Conforti R, Cirillo M, Sardaro A, Caiazzo G, Negro A, Paccone A, Sacco R, Sparaco M, Gallo A, Lavorgna L, Tedeschi G, Cirillo S. Dilated perivascular spaces and fatigue: is there a link? Magnetic resonance retrospective 3Tesla study. Neuroradiology. 2016; 58:859–66. 10.1007/s00234-016-1711-0 27423658

[32] Groeschel S, Brockmann K, Hanefeld F. Virchow-Robin spaces on magnetic resonance images of children with adrenoleukodystrophy. Eur J Paediatr Neurol. 2007; 11:142–45. 10.1016/j.ejpn.2006.11.014 17254818

[33] Doubal FN, MacLullich AM, Ferguson KJ, Dennis MS, Wardlaw JM. Enlarged perivascular spaces on MRI are a feature of cerebral small vessel disease. Stroke. 2010; 41:450–54. 10.1161/STROKEAHA.109.564914 20056930

[34] Potter GM, Doubal FN, Jackson CA, Chappell FM, Sudlow CL, Dennis MS, Wardlaw JM. Enlarged perivascular spaces and cerebral small vessel disease. Int J Stroke. 2015; 10:376–81. 10.1111/ijs.12054 23692610 PMC4463944

[35] Arba F, Quinn TJ, Hankey GJ, Lees KR, Wardlaw JM, Ali M, Inzitari D, and VISTA Collaboration. Enlarged perivascular spaces and cognitive impairment after stroke and transient ischemic attack. Int J Stroke. 2018; 13:47–56. 10.1177/1747493016666091 27543501

[36] Duker AP, Espay AJ. Parkinsonism associated with striatal perivascular space dilation. Neurology. 2007; 68:1540. 10.1212/01.wnl.0000261483.49248.b8 17470760

[37] Conforti R, Sardaro A, Negro A, Caiazzo G, Paccone A, De Micco R, Cirillo S, Tessitore A. Dilated Virchow-Robin space and Parkinson’s disease: A case report of combined MRI and diffusion tensor imaging. Radiol Case Rep. 2018; 13:871–77. 10.1016/j.radcr.2018.05.011 29988793 PMC6031236

[38] Park YW, Shin NY, Chung SJ, Kim J, Lim SM, Lee PH, Lee SK, Ahn KJ. Magnetic Resonance Imaging-Visible Perivascular Spaces in Basal Ganglia Predict Cognitive Decline in Parkinson’s Disease. Mov Disord. 2019; 34:1672–79. 10.1002/mds.27798 31322758

[39] Pesce C, Carli F. Allometry of the perivascular spaces of the putamen in aging. Acta Neuropathol. 1988; 76:292–94. 10.1007/BF00687778 3213433

[40] Bouvy WH, Biessels GJ, Kuijf HJ, Kappelle LJ, Luijten PR, Zwanenburg JJ. Visualization of perivascular spaces and perforating arteries with 7 T magnetic resonance imaging. Invest Radiol. 2014; 49:307–13. 10.1097/RLI.0000000000000027 24473365

[41] Shen T, Yue Y, Zhao S, Xie J, Chen Y, Tian J, Lv W, Lo CZ, Hsu YC, Kober T, Zhang B, Lai HY. The role of brain perivascular space burden in early-stage Parkinson's disease. NPJ Parkinsons Dis. 2021; 7:12. 10.1038/s41531-021-00155-0 33547311 PMC7864928

[42] Zong X, Park SH, Shen D, Lin W. Visualization of perivascular spaces in the human brain at 7T: sequence optimization and morphology characterization. Neuroimage. 2016; 125:895–902. 10.1016/j.neuroimage.2015.10.078 26520772

[43] Banerjee G, Kim HJ, Fox Z, Jäger HR, Wilson D, Charidimou A, Na HK, Na DL, Seo SW, Werring DJ. MRI-visible perivascular space location is associated with Alzheimer’s disease independently of amyloid burden. Brain. 2017; 140:1107–16. 10.1093/brain/awx003 28335021

[44] Hirabuki N, Fujita N, Fujii K, Hashimoto T, Kozuka T. MR appearance of Virchow-Robin spaces along lenticulostriate arteries: spin-echo and two-dimensional fast low-angle shot imaging. AJNR Am J Neuroradiol. 1994; 15:277–81. 8192073 PMC8334624

[45] Pantoni L. Cerebral small vessel disease: from pathogenesis and clinical characteristics to therapeutic challenges. Lancet Neurol. 2010; 9:689–701. 10.1016/S1474-4422(10)70104-6 20610345

[46] Bath PM, Wardlaw JM. Pharmacological treatment and prevention of cerebral small vessel disease: a review of potential interventions. Int J Stroke. 2015; 10:469–78. 10.1111/ijs.12466 25727737 PMC4832291

[47] Jessen NA, Munk AS, Lundgaard I, Nedergaard M. The Glymphatic System: A Beginner’s Guide. Neurochem Res. 2015; 40:2583–99. 10.1007/s11064-015-1581-6 25947369 PMC4636982

[48] Malek N, Lawton MA, Swallow DM, Grosset KA, Marrinan SL, Bajaj N, Barker RA, Burn DJ, Hardy J, Morris HR, Williams NM, Wood N, Ben-Shlomo Y, Grosset DG, and PRoBaND Clinical Consortium. Vascular disease and vascular risk factors in relation to motor features and cognition in early Parkinson’s disease. Mov Disord. 2016; 31:1518–26. 10.1002/mds.26698 27324570 PMC5082556

[49] Nutt JG, Horak FB, Bloem BR. Milestones in gait, balance, and falling. Mov Disord. 2011; 26:1166–74. 10.1002/mds.23588 21626560

[50] Wan Y, Hu W, Gan J, Song L, Wu N, Chen Y, Liu Z. Exploring the association between Cerebral small-vessel diseases and motor symptoms in Parkinson’s disease. Brain Behav. 2019; 9:e01219. 10.1002/brb3.1219 30815987 PMC6456802

[51] Fling BW, Cohen RG, Mancini M, Nutt JG, Fair DA, Horak FB. Asymmetric pedunculopontine network connectivity in parkinsonian patients with freezing of gait. Brain. 2013; 136:2405–18. 10.1093/brain/awt172 23824487 PMC3722352

[52] Bartels AL, de Jong BM, Giladi N, Schaafsma JD, Maguire RP, Veenma L, Pruim J, Balash Y, Youdim MB, Leenders KL. Striatal dopa and glucose metabolism in PD patients with freezing of gait. Mov Disord. 2006; 21:1326–32. 10.1002/mds.20952 16721756

[53] Snijders AH, Leunissen I, Bakker M, Overeem S, Helmich RC, Bloem BR, Toni I. Gait-related cerebral alterations in patients with Parkinson’s disease with freezing of gait. Brain. 2011; 134:59–72. 10.1093/brain/awq324 21126990

[54] Bartels AL, Leenders KL. Brain imaging in patients with freezing of gait. Mov Disord. 2008 (Suppl 2); 23:S461–67. 10.1002/mds.21912 18668627

[55] Katzen HL, Levin BE, Weiner W. Side and type of motor symptom influence cognition in Parkinson’s disease. Mov Disord. 2006; 21:1947–53. 10.1002/mds.21105 16991155

[56] van der Hoorn A, Burger H, Leenders KL, de Jong BM. Handedness correlates with the dominant Parkinson side: a systematic review and meta-analysis. Mov Disord. 2012; 27:206–10. 10.1002/mds.24007 21994149

[57] Wright WG, Gurfinkel VS, King LA, Nutt JG, Cordo PJ, Horak FB. Axial kinesthesia is impaired in Parkinson’s disease: effects of levodopa. Exp Neurol. 2010; 225:202–09. 10.1016/j.expneurol.2010.06.016 20599976 PMC3052408

[58] Tessitore A, Amboni M, Esposito F, Russo A, Picillo M, Marcuccio L, Pellecchia MT, Vitale C, Cirillo M, Tedeschi G, Barone P. Resting-state brain connectivity in patients with Parkinson’s disease and freezing of gait. Parkinsonism Relat Disord. 2012; 18:781–87. 10.1016/j.parkreldis.2012.03.018 22510204

[59] Horak FB, Nutt JG, Nashner LM. Postural inflexibility in parkinsonian subjects. J Neurol Sci. 1992; 111:46–58. 10.1016/0022-510x(92)90111-w 1402997

[60] Horak FB, Frank J, Nutt J. Effects of dopamine on postural control in parkinsonian subjects: scaling, set, and tone. J Neurophysiol. 1996; 75:2380–96. 10.1152/jn.1996.75.6.2380 8793751

[61] Kim KW, MacFall JR, Payne ME. Classification of white matter lesions on magnetic resonance imaging in elderly persons. Biol Psychiatry. 2008; 64:273–80. 10.1016/j.biopsych.2008.03.024 18471801 PMC2593803

[62] Griffanti L, Jenkinson M, Suri S, Zsoldos E, Mahmood A, Filippini N, Sexton CE, Topiwala A, Allan C, Kivimäki M, Singh-Manoux A, Ebmeier KP, Mackay CE, Zamboni G. Classification and characterization of periventricular and deep white matter hyperintensities on MRI: A study in older adults. Neuroimage. 2018; 170:174–81. 10.1016/j.neuroimage.2017.03.024 28315460

[63] Hughes AJ, Daniel SE, Kilford L, Lees AJ. Accuracy of clinical diagnosis of idiopathic Parkinson’s disease: a clinico-pathological study of 100 cases. J Neurol Neurosurg Psychiatry. 1992; 55:181–84. 10.1136/jnnp.55.3.181 1564476 PMC1014720

[64] Giladi N, Shabtai H, Simon ES, Biran S, Tal J, Korczyn AD. Construction of freezing of gait questionnaire for patients with Parkinsonism. Parkinsonism Relat Disord. 2000; 6:165–70. 10.1016/s1353-8020(99)00062-0 10817956

[65] Tomlinson CL, Stowe R, Patel S, Rick C, Gray R, Clarke CE. Systematic review of levodopa dose equivalency reporting in Parkinson’s disease. Mov Disord. 2010; 25:2649–53. 10.1002/mds.23429 21069833

[66] Maclullich AM, Wardlaw JM, Ferguson KJ, Starr JM, Seckl JR, Deary IJ. Enlarged perivascular spaces are associated with cognitive function in healthy elderly men. J Neurol Neurosurg Psychiatry. 2004; 75:1519–23. 10.1136/jnnp.2003.030858 15489380 PMC1738797

[67] Scheltens P, Barkhof F, Leys D, Pruvo JP, Nauta JJ, Vermersch P, Steinling M, Valk J. A semiquantative rating scale for the assessment of signal hyperintensities on magnetic resonance imaging. J Neurol Sci. 1993; 114:7–12. 10.1016/0022-510x(93)90041-v 8433101

